# Investigating the Neural Correlates of Voice versus Speech-Sound Directed Information in Pre-School Children

**DOI:** 10.1371/journal.pone.0115549

**Published:** 2014-12-22

**Authors:** Nora Maria Raschle, Sara Ashley Smith, Jennifer Zuk, Maria Regina Dauvermann, Michael Joseph Figuccio, Nadine Gaab

**Affiliations:** 1 Laboratories of Cognitive Neuroscience, Division of Developmental Medicine, Department of Developmental Medicine, Boston Children's Hospital, Boston, Massachusetts, United States of America; 2 Harvard Medical School, Boston, Massachusetts, United States of America; 3 Psychiatric University Clinics Basel, Department of Child and Adolescent Psychiatry, Basel, Switzerland; 4 Harvard Graduate School of Education, Cambridge, Massachusetts, United States of America; University of Barcelona, Spain

## Abstract

Studies in sleeping newborns and infants propose that the superior temporal sulcus is involved in speech processing soon after birth. Speech processing also implicitly requires the analysis of the human voice, which conveys both linguistic and extra-linguistic information. However, due to technical and practical challenges when neuroimaging young children, evidence of neural correlates of speech and/or voice processing in toddlers and young children remains scarce. In the current study, we used functional magnetic resonance imaging (fMRI) in 20 typically developing preschool children (average age  = 5.8 y; range 5.2–6.8 y) to investigate brain activation during judgments about vocal identity versus the initial speech sound of spoken object words. FMRI results reveal common brain regions responsible for voice-specific and speech-sound specific processing of spoken object words including bilateral primary and secondary language areas of the brain. Contrasting voice-specific with speech-sound specific processing predominantly activates the anterior part of the right-hemispheric superior temporal sulcus. Furthermore, the right STS is functionally correlated with left-hemispheric temporal and right-hemispheric prefrontal regions. This finding underlines the importance of the right superior temporal sulcus as a temporal voice area and indicates that this brain region is specialized, and functions similarly to adults by the age of five. We thus extend previous knowledge of voice-specific regions and their functional connections to the young brain which may further our understanding of the neuronal mechanism of speech-specific processing in children with developmental disorders, such as autism or specific language impairments.

## Introduction

The human voice is omnipresent in our lives, conveying both linguistic and extra-linguistic information and playing an integral role in our daily interactions. In addition to delivering language content, the human voice conveys rich acoustic information crucial for speaker identification, such as the fundamental frequency of the speaker's voice and the spectral formants produced through modification of the vocal tract that characterize individual vowels and consonants [1]–[2]. The latter carries the prosodic features of communication [3] as well as emotional tone [4], and additionally provides cues to determine age [5] and gender [6]–[7]. Behavioral research has, for example, shown that infants as young as eight months old are able to recognize male and female voices [8]. Voice perception carries many different socially relevant features, demanding complex processes from our brain. It has been proposed that the cerebral processing of vocal information (e.g., speaker identity or affect) may be organized in interacting, but functionally dissociable pathways [9]–[11]. Neuropsychological evidence [12]–[13] suggests that voice and speech-sound directed information may be processed differently.

Adults show a preference for general speech processing in bilateral temporal brain regions, particularly in superior temporal gyrus (STG) and superior temporal sulcus (STS; [12]). Using neuroimaging techniques such as functional magnetic resonance imaging (fMRI), electroencephalography (EEG) or positron emission tomography (PET), a human-specific voice-processing region has been suggested in the upper bank of the right STS [9], [14]–[16]. It is of note that some studies have identified voice processing areas in bilateral STS [11], [17]–[18]. However, the vast majority of publications report right-hemispheric neuronal activation within the anterior part of the right STS, specifically when processing human vocal sounds [9], [15], [17]. The idea of voice-specific sound processing in humans is supported by studies comparing the neuronal correlates of vocal sounds to activation patterns evoked by frequency modulated tones [19] or by comparing human vocal sounds to those produced by animals [20]–[21]. Although parts of the STS are activated in response to both vocal and non-vocal sounds (e.g., environmental or artificial sounds), vocal sounds produce greater neural responses than non-vocal sounds in most voice selective regions of the brain [7], [16], [17], [22]. Furthermore, fMRI evidence shows that activity in the right STS is greater when subjects perform a voice identity task (hearing several speakers say the same word) as opposed to speech-sound specific tasks [10], [15], [19], [23], providing evidence for the involvement of the anterior portion of the right STS in processing voice identity.

The majority of research on the neural mechanisms of speech and voice processing has been conducted in adult participants; however, infant studies implementing passive listening experiments in the first years of life have been reported. Behavioral research using the head-turn procedure in newborns could, for example, show that newborns prefer human to non-human sounds, as well as prosodic to non-prosodic speech [24]. Neuroimaging methods, such as near infrared spectroscopy (NIRS) or fMRI, have shed light on hemispheric specialization for speech, but provided mixed results. While some studies report increased activation during human speech processing within right temporoparietal brain regions (e.g., [25]; NIRS during human speech compared to flattened speech sounds in three month olds), others have suggested a left-hemispheric lateralization of human speech in newborns (e.g., [26]; optical topography during speech processing in newborns). Left-hemispheric lateralization of speech processing is further supported by fMRI evidence in three month old infants [27]–[28].

Similar to adult studies, the anterior STS in infants was observed to be critically involved during passive listening to human speech (e.g., comparing non-speech human emotional sounds versus familiar non-voice background noises; [29]). However, unlike in adults, infants did not show different activation patterns when processing forward, as opposed to backward, speech, leading the authors to conclude that the precursors to adult-like cortical language processing were observable, but most likely not yet specialized [28], [30]. In line with this finding, Grossmann and colleagues [3] reported that four month-old infants did not display increased activation within bilateral superior temporal cortices when contrasting human speech with non-vocal sounds. However, at seven months, distinctive activation patterns can be observed during human voice and artificial sounds processing in left and right superior temporal cortex, comparable to results seen in adults [3]. To summarize, research so far has provided mixed results regarding the activation pattern representing speech processing in infancy (e.g., [25], [28]). However, the neuronal basis for speech is somehow similar to that of adults, increasingly so with age. Improved specialization, as observed by distinct neuronal activation to human speech sounds compared to a control condition (backward speech in [28]; environmental sounds in [3]), takes place between four and seven months- notably at a time when speech content is still fairly irrelevant [3].

Though there is evidence for the neuronal basis of passive speech and vocal information processing in infants, as well as plentiful studies in adults, a gap in neuroimaging studies exists with toddler and preschool-aged participants. However, technical improvements and increasingly more elaborate child-friendly neuroimaging protocols allow for the extension of studies into younger age ranges [31]–[33]. This is of utmost importance since previous developmental neuroimaging work has demonstrated that there are significant differences between children and adults in regard to brain structure and function (e.g., [34]–[39]); thus assumptions of a static model of the human brain are outdated [40]. Moreover, even though studies in infancy are able to inform about crucial aspects of brain organization and development closely after birth, a response-related cognitive functional neuroimaging task including behavioral feedback is not yet possible, and thus assumptions based on findings from research in sleeping infants may not easily be applied to waking children. Finally, evidence that the neuronal circuits for specific aspects of speech processing (e.g., phoneme discrimination) undergo changes in the first year of life to attune to native language sounds underscores the need for evaluation of the young brain before and after the onset of speech production [41]. To our knowledge, only one exemplary study has examined electrophysiological correlates of voice processing in a study of four and five year-olds. Comparing voices to environmental sounds resulted in an early measurable deflection (within 60 ms of onset) at right fronto-temporal sites, evidence for a right-lateralized response to voices in children [42]. The authors have suggested that this response may reflect activation of temporal voice areas in children.

To summarize, there is a general consensus regarding the neural location and functional correlates of voice processing in adults [10]–[11], [17], [43]–[46] and there is evidence for an early manifestation of this skill [3]. However, the precise anatomical localization, neuronal correlates and functional connectivity of voice processing in pre-school or school-aged children remains less well-understood. While infant research has explored activation in response to ‘normal’ forward speech compared to speech presented backwards, as well as between vocal and non-vocal sounds, few studies with pre-school aged children have investigated activation evoked specifically by varying aspects of native speech (for example, vocal pitch as compared to speech-sound specific content). Therefore, the current study employed whole brain fMRI in 20 typically developing pre-school children. The objective of the present work was to investigate cortical response to two auditory tasks in five year-old participants. The experimental task employed was voice directed (voice matching (VM): ‘Is it the same person/voice talking or a different person?’), while the second task was a speech-sound directed, phonological processing task (first sound matching (FSM): ‘Do both words begin with the same first sound?’). The same two voices, one male and one female, were maintained throughout both tasks. In a comparable study in adults, Von Kriegstein and colleagues [9] demonstrated that the right anterior STS is activated during tasks requiring voice processing but not when content directed processing was targeted. A follow-up fMRI study in adults [10] was furthermore able to identify distinct but interacting areas along the right STS responding to acoustic information stimuli, task demand and speaker-familiarity independently. Furthermore, previous evidence from fMRI studies suggests the bilateral STS to be crucial for processing human voices compared to non-speech sounds [17]. However, it has been suggested that the right STS alone is significantly more activated for processing nonverbal components of human speech (e.g., voice identity unrelated to verbal content; [9]–[10]). Therefore, we hypothesize that the right STS will be recruited during the voice but not speech-sound directed task in pre-schoolers, similar to the neuronal pattern observed in adult participants. To test this hypothesis, we explicitly investigate patterns of neural activation as well as functional connectivity during a voice identification task in right and left-hemispheric STS regions.

## Methods

### Ethics Statement

This study and its consent procedures were approved by the Boston Children's Hospital Committee of Clinical Investigation (CCI). Written informed consent on behalf of the child participants was obtained from guardians (first degree relatives). Furthermore, research team members obtained verbal assent from child participants. Verbal assent, and not written consent, was obtained from child participants due to their young age (average age 5.8 y; children were non-readers and could not write yet).

### Participants

Twenty healthy, native English speaking children (average age at the time of imaging: 5.8 years, age range 5.2 to 6.8 years) were included in the present analysis. Nineteen children were right handed, whereas for one child handedness could not be indicated yet (labeled as ambidextrous). All children were physically healthy with no history of any neurological or psychological disorders, head injuries, poor vision or poor hearing. All children scored within the normal or above-average range on verbal and non-verbal IQ (Kaufman Brief Intelligence Test, 2^nd^ edition [47]; **Table 1**). All children in the current study are part of the Boston Longitudinal Study of Dyslexia (BOLD) at the Boston Children's Hospital, a study that aims to investigate neural and behavioral characteristics of typical developing children compared to those with a familial risk for developmental dyslexia. Participants are invited for one behavioral and one neuroimaging session, including three functional experiments and structural image acquisition. The results presented here are from a subgroup of typically developing children at the first time point of neuroimaging (all children that had useful data obtained during the voice- and speech-sound-directed task were included).

**Table 1:** Behavioral Group Characteristics.

		Mean ± SD
N		
Age (in months/psychometrics session)		66.5±4.3
Age (in months/imaging session)		70.5±6.2
Behavioral Measures		
CELF	Core Language^a^	109.1±9.5
	Receptive Language^a^	108.0±11.1
	Expressive Language^a^	108.5±10.0
	Language Content^a^	108.6±11.2
	Language Structure^a^	108.4±9.7
CTOPP	Elision a	10.5±2.5
	Blending a	11.5±1.6
	Non-Word Repetition a	10.2±2.2
RAN	Objects b	104.1±11.8
	Colors a	103.6±13.9
VATT	Inflection c	25.8±8.5
	Repetition c	38.5±1.9
KBIT	Verbal Ability b	110.1±8.3
	Non-Verbal Ability b	101.9±11.8
**Socioeconomic Status** (see also S1 Table)		
Parental Education d ^,^ e		6.2±0.8
Income		
(total family income for last 12 months) f		11.9

Measures (standard scores are reported).

a 19 FHD- (One child did not finish all testing).

b 18 FHD- (Two children did not finish all testing).

c 17 FHD- (Three children did not finish all testing).

d 16 FHD- (Four children did not finish all testing).

e Parental Education scores are calculated according to the 7-point Hollingshead Index Educational Factor.

Scale, summed for husband and wife and divided by two (Hollingshead, 1975).

f Scale where 10–5,000 $, 2 = 5,000–11,999 $, 3 = 12,000–15,999 $, 4 = 16,000–24,999 $, 5 = 25,000–34,999 $, 6 = 35,000–49,900 $, 7 = 50,000–74,999 $, 8 = 75,000–99,999 $, 9 = 100,000+$, 10 =  Don't know, 11 =  No Response.

### Behavioral Group Characteristics

Participants were characterized with a test battery of standardized assessments examining language and pre-reading skills, such as expressive and receptive vocabulary (Clinical Evaluation of Language Fundamentals (CELF Preschool 2nd edition; [48]), phonological processing (Comprehensive Test of Phonological Processing (CTOPP); [49] and Verb Agreement and Tense Test (VATT; [50]) and rapid automatized naming (Rapid Automatized Naming Test; [51]). Additionally, all participating families were given a socioeconomic background questionnaire (questions adapted from the MacArthur Research Network: http://www.macses.ucsf.edu/Default.htm; for a complete overview of SES questions see **S1 Table**) and were asked questions regarding language development (see **S2 Table**). All children were assessed for verbal and non-verbal IQ (KBIT average verbal IQ  = 110.1±8.3; average non-verbal IQ = 101.9±11.8) and socioeconomic status (SES). Behavioral testing and imaging were performed on different days, however, there were no more than ±42 days between the two sessions on average (less than 1.5 months).

### fMRI - Task Procedure

Each child performed two consecutive fMRI runs with identical designs, including timing and duration. One run consisted of a voice directed task (voice matching (VM): ‘Is it the same person/voice talking or a different person?’), while the other run consisted of a speech-sound directed, phonological processing task (first sound matching (FSM): ‘Do both words begin with the same first sound?’). The same two voices, one male and one female, were maintained throughout the tasks. The female voice had an average fundamental frequency of 218 Hz and was significantly higher in the test items [*t*(54) = 15, p<.001] than the male voice (average fundamental frequency of 131 Hz.) The order of the two runs was pseudo-randomized (participants used a dice to determine the order).The FSM and VM tasks were presented in two separate runs in order to reduce task demands (e.g., task switching) for the young participants, based on previous experience carrying out neuroimaging studies in young populations (see also [32]–[33], [52]). During the ***VM task*** all children listened to two subsequently presented common object words spoken in a female or male voice via MR-compatible noise-reducing headphones (two seconds per word). During both runs, corresponding pictures were presented on the screen simultaneously in order to engage the children and to reduce working memory demands. The object words were followed by the presentation of a question mark, also displayed for two seconds. Using two child-friendly buttons placed on either side of the participant, children were asked to indicate via button-press whether the voice matched for the two words presented. This task was contrasted with a rest condition, during which the children were required to look at a fixation cross for the duration of the block. During ***the FSM task***, participants were again asked to listen to two common object words, spoken in a female or male voice. Participants indicated via button press whether the two words presented started with the same first sound (e.g., *bed* and *belt*; “yes”, or not (e.g., *bird* and *ant*; “no”, for details see also [52]). This task was again contrasted with a rest condition. Reaction time was measured from the start of the second word on and the response window lasted until the start of the consecutive trial for both tasks.

To avoid repetition effects (e.g., [53]), different word lists were created for the VM and FSM tasks. However, all words between the two runs were kept as comparable as possible by matching the two word lists for age of acquisition (e.g., when an average child recognizes a certain word; all words used here are recognized before 4 years of age by typically developing children), Brown verbal frequency, concreteness, imagery, numbers of letters, numbers of phonemes and numbers of syllables (MRWC Psycholinguistic and the IPNP Database; http://www.psy.uwa.edu.au/mrcdatabase/uwa_mrc.html and http://crl.ucsd.edu/~aszekely/ipnp/). All pictures were adapted from the standardized Snodgrass Picture System [54]. The same number of trials for VM and FSM matches were included in each run (for further task descriptions see [52]). A sparse temporal sampling design allowed for the presentation of the auditory stimuli without scanner background noise interference [55]–[57]. A total of seven blocks of VM/FSM and seven blocks of rest condition with an overall duration of 336 s seconds for each run were employed. Each block lasted 24 seconds and each block contained four trials. During the experimental and control tasks, 50% of the words were spoken in a male/female voice and 50% of all items matched regarding their first sound. The order of trials within a block was randomized, but kept constant across participants.

Each child underwent extensive preparation and training in the mock MR scanner area before the actual neuroimaging session. Participants were familiarized with the voice and speech-sound directed task prior to the neuroimaging session using unique practice items. Instructions for each task were presented in separate short videos, which were shown in the MR scanner area and repeated prior to actual scanning. To reduce movement during the scanning procedure, cushions were used to stabilize the head and response buttons were placed at arm's length on each side of the child. A member of the research team observed the child during in-scanner performance and provided a tactile reminder to stay still during the session if needed (for a detailed description of the training protocol see [32]–[33]).

### fMRI - Acquisition and Analysis

For each run (experimental and control task), 56 functional whole-brain images were acquired with a 32 slice EPI interleaved acquisition on a SIEMENS 3T Trio MR scanner including the following specifications: TR 6000 ms; TA 1995 ms; TE 30 ms; flip angle 90°; field of view 256 mm; voxel size 3×3×4 mm, slice thickness 4 mm. Prior to the start of the first block, additional functional images were obtained and later discarded to allow for T1 equilibration effects. Stimuli were presented using Presentation software (Version 0.70, www.neurobs.com). The complete imaging session included 2 additional functional imaging tasks; actual scan time per task was 12 minutes each.

Image processing and analyses were carried out using SPM5 (www.fil.ion.ucl.ac.uk/spm) executed in MATLAB (Mathworks, Natick, MA). Prior to statistical analysis, we first adjusted for movement artifacts within the acquired fMRI time series by realigning all images using a least squares approach to the first image (after discarding the first images to allow for T1 equilibration effects). In a second step, all images were spatially normalized into standard space, as defined by the ICBM, NIH-20 project [58]. It is to note that no customized child template was used and that consequent reports of coordinates and activation pattern are interpreted with caution due to the brain size differences of adults and children. Finally, all images were smoothed with an 8 mm full width at half maximum (FWHM) isotropic kernel to remove noise and effects due to residual differences in functional and structural anatomy during inter-subject averaging (SPM5). Due to the age of participants, a rigorous procedure for artifact detection was implemented. We used the art-imaging toolbox (http://www.nitrc.org/projects/artifact_detect) to visualize motion, plot potential movement artifacts and review analysis masks for each subject. Upon visual inspection of all raw images, preprocessed images were used to create an explicit mask excluding potential artifactual brain volumes from the explicit mask through the art-imaging toolbox for each child. The art-imaging toolbox was then used to plot differences in motion between consecutive images and to review artifactual time-points: First, we identified all images that exceeded a movement threshold of 2 mm and a rotation threshold of 0.05 mm and checked that the analysis mask without said images contained all voxels (this step is necessary to ensure that there are no remaining outliers in the images within the defined threshold). Every image exceeding this threshold was then visually inspected, and movement and outlier regressors were added to the general linear model. Furthermore, volumes containing visible artifacts were regressed out and not modeled in further analyses. Prior to first level analysis, we ensured that the explicit mask was complete (inclusion of all brain voxels). The general linear approach implemented in SPM5 was used to analyze the data in a block design for each subject. Movement regressors were modeled as cofounds within the general linear model and explicit masking was performed during each subject's first level analysis to ensure inclusion of each voxel of the analysis mask. Contrast images (One sample t-tests) for ‘VM>Rest’, ‘FSM>Rest’, ‘FSM>VM’ (contend directed contrast) and ‘VM>FSM’ (voice directed contrast) were obtained for the whole group of children. Because of the lower signal-to-noise ratio in pediatric compared to adult samples and the relatively high inter-individual variance in pediatric datasets (e.g. [95]), results are reported at a threshold of p<0.005 with a cluster extent threshold of k = 10, as similarly employed by other pediatric studies (e.g. [52], [96]).

### Region of Interest (ROI) Analysis

Previous research has shown involvement of the right anterior STS during voice processing, particularly during the analysis of extra-linguistic features of speech [9]. To investigate the role of the right anterior STS further, we defined an ROI for the anterior part of the right STS based on evidence in adults [15] (4 mm-sphere at Talairach coordinates of peak: 58,2,-8) using the MarsBaR toolbox [95]. Using the MarsBaR transformation function, we flipped this right hemispheric ROI to create a left-hemispheric analogue (left STS ROI). Mean parameter estimates were extracted for the two resulting regions of interest for the conditions ‘VM>Rest’, ‘FSM>Rest’ and ‘VM<FSM’ and ‘VM>FSM’ to further characterize the involvement of these regions during voice or speech-sound directed processing. A paired two-samples T-Test was employed to test for lateralization effects during ‘VM>FSM’.

### Functional Connectivity MRI (fcMRI) Analysis

A post-hoc seed-to-voxel bivariate correlation analysis was performed using the MATLAB-based custom software package CONN [59]. Additional fcMRI analysis-specific preprocessing steps included temporal band-pass filtering and regression of nuisance variables including signal from white matter and cerebrospinal fluid. Source seeds, defined as the right and left-hemispheric STS (as extracted for the ROI analysis) were specified as multiple seeds. Seed-based correlation maps were created by extracting the residual BOLD time series from the seed regions, which were followed by Pearson's correlation coefficients between the average time series of each seed and the time series of all other voxels. Seed-to-voxel correlation maps for the right and left STS for each subject and the condition ‘VM>FSM’ were created. For the second-level seed-based fcMRI analysis, results are reported at a significance level of p<0.005, uncorrected, and an ET of 50 voxels.

### In-Scanner Performance

Button presses were recorded during voice and speech-sound directed speech processing tasks. Participants' in-scanner performance was closely monitored to ensure participation (for details see [32]–[33]). Children were instructed to indicate their answer as soon as they saw a question mark appear on the screen (after the presentation of the second word; for task design and figure see also [52]), and responses were collected until the first word of the second trial was presented. Due to the nature of the task, however, children were able to form their judgment soon after the start of the presentation of the second word. Children were allowed to correct their response until the first word of a consecutive trial was presented. Task accuracy was calculated. The current study employs a block design; therefore trials with in-scanner performance errors were included in the analysis.

## Results

### Demographics and Behavioral Group Characteristics

Demographics and behavioral group characteristics are listed in **Table 1**. All children scored average or above average on standardized tests of pre-reading and language skills, including expressive and receptive language skills, phonological processing, rapid automatized naming, and verbal and non-verbal IQ.

### In-Scanner Performance

Due to a technical problem, the behavioral response for one child is missing. Since the child's performance during training indicated that the child understood the tasks, we decided to include the participant in consequent analyses. Behavioral responses given by button presses during in-scanner performance indicate a recognition rate for the speaker identification task (VM) of 79.8% (average raw score of 22.3±4.6 out of N = 28), 13.7% incorrect (average raw score of 3.8±4.2) and 6.6% misses (average raw score of 1.8±1.7), and a recognition rate of 73.1% (average raw score of 20.5±5.1), 18.6% incorrect (average raw score of 5.2±4.1) and 8.3% misses (average raw score of 2.3±2.5) during the speech-sound directed task (FSM). Paired two sample *t*-tests showed that there was no difference in the amount of correct responses between voice versus speech-sound directed tasks (p>0.05). Furthermore, no difference in reaction times were observed between the two tasks (p>0.05; VM RT = 2338.1 ms/FSM RT = 2305.4 ms).

### fMRI results

**Figure 1:**
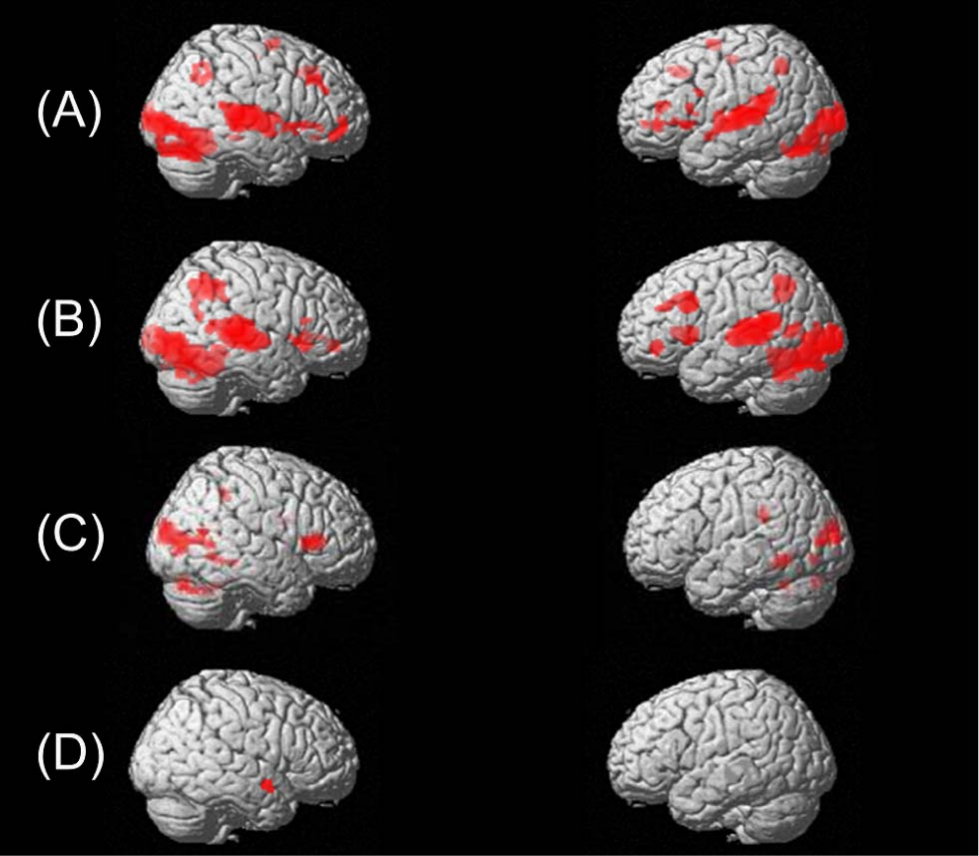
Neuronal activation patterns during voice or speech-sound directed information processing. Cerebral regions activated when attending to (**A**) speakers voice (‘VM> Rest’) or (**B**) speech sounds (‘FSM> Rest’). Brain regions activated when attending more on speech sounds of spoken object words than speaker voice (**C**; ‘VM <FSM’) and regions activated when attending more to speaker voice than speech sounds of spoken object words (**D**; ‘VM> FSM’ (p<0.005; k = 10).

Whole-brain analysis revealed increased activation for both voice directed (voice matching (VM)) and speech-sound directed processing (first sound matching (FSM)) in brain regions including bilateral middle occipital/fusiform gyrus, middle/inferior frontal gyrus, superior/middle temporal gyrus and inferior/superior parietal lobe when contrasted against rest (**Fig. 1A,B**; **Table 2**). Focusing more on the initial speech sounds than speaker's voice (VM<FSM) activated a predominantly left-hemispheric language network including fusiform gyrus, inferior occipital/lingual and middle occipital gyrus (**Fig. 1C** **;** **Table 2**; for an in depth discussion regarding the greater activation during the FSM task when compared to the VM task, see also [52]). However, when focusing more on speaker identification compared to initial speech sounds (VM>FSM), brain activation occurred within the right anterior middle/superior temporal gyrus (**Fig. 1D** **;** **Table 2**).

**Table 2:** Main cortical peak activations for speech sounds or speaker voice compared to rest (‘FSM>Rest’ and ‘VM>Rest’), or speech sounds vs. speaker voice (‘VM<FSM’ and ‘VM>FSM’).

Region		x	y	z	Z	Size, voxels	Region		x	y	z	Z	Size, voxels
**Attending to speakers voice (VM> Rest)**							**Attending to speech sounds (FSM> Rest)**						
*Occipital Lobe*							*Occipital Lobe*						
	Middle Occipital/Fusiform Gyrus (R)	36	−72	−24	5.3	2482		Middle Occipital/Fusiform Gyrus (R)	36	−48	−18	5.1	2459
	Middle Occipital/Fusiform Gyrus (L)	−38	−72	−22	4.9	1286		Middle Occipital/Fusiform Gyrus (L)	−24	−100	−2	5.1	2814
	Lingual Gyrus (R)	8	−82	16	4.1	43							
*Frontal Lobe*							*Frontal Lobe*						
	Inferior Frontal Gyrus (L)	−54	16	14	3.1	19		Inferior Frontal Gyrus/Insula (R)	40	22	16	3.7	36
	Inferior Frontal Gyrus (L)	−50	8	26	3.1	26		Inferior Frontal Gyrus (R)	52	22	−4	3.4	109
	Inferior Frontal/Superior Temporal Gyrus (R)	54	20	−2	3.4	107		Inferior/Middle Frontal Gyrus (L)	−50	18	28	4.3	292
	Inferior/Middle Frontal Gyrus (L)	−46	20	0	4.4	146		Insula/Extra Nuclear (L)	−26	22	6	3.9	145
	Medial Frontal Gyrys (L)	−12	−20	52	3.8	32		Insula/Inferior Frontal Gyrus (R)	36	22	6	3.4	42
	Medial Frontal/Cingulate Gyrus (L/R)	2	26	44	3.2	153		Insula/Precentral/Inferior Frontal Gyrus (L)	−44	14	4	3.5	166
	Middle Frontal Gyrus (R)	42	46	−8	3.6	289		Middle Frontal Gyrus (L)	−46	40	−6	3.2	88
	Middle Frontal Gyrus (R)	46	28	36	3.3	107		Middle/Medial Frontal Gyrus (R)	20	44	−6	3.4	71
	Middle/Inferior Frontal Gyrus (L)	−34	40	16	3.2	83							
	Middle/Superior Frontal Gyrus (L)	−28	52	0	2.9	30							
	Superior/Medial Frontal Gyrus (L/R)	−2	−2	66	3.2	91							
*Temporal Lobe*							*Temporal Lobe*						
	Superior Temporal Gyrus (R)	−52	−20	6	5.0	1631		Middle/Superior Temporal Gyrus (R)	64	−28	10	5.4	1531
	Superior/Middle Temporal Gyrus (R)	58	−26	8	4.7	1531		Middle/Superior Temporal Gyrus (L)	−60	−42	16	5.7	1620
*Parietal Lobe*							*Parietal Lobe*						
	Inferior Parietal Lobe/Supramarginal Gyrus (R)	50	−52	42	2.9	31		Inferior/Superior Parietal Lobe (R)	44	−46	42	4.1	681
	Inferior Parietal Lobe (R)	34	−62	40	3.2	99		Inferior/Superior Parietal Lobe (L)	−26	−58	44	3.7	385
	Inferior/Superior Parietal Lobe/Precuneus (L)	−28	−54	50	3.8	95		Precuneus (R)	14	−62	48	3.0	21
**Attending speech sounds compared to speaker voice (VM <FSM)**							**Attending speaker voice compared to speech sounds (VM> FSM)**						
*Occipital Lobe*							*Temporal Lobe*						
	Fusiform Gyrus (L)	−38	−48	−10	3.3	84		Middle/Superior Temporal Gyrus (R)	60	2	−14	2.9	20
	Inferior Occipital/Lingual Gyrus (L)	−32	−76	−6	2.9	17							
	Middle Occipital Gyrus (L)	30	−68	8	2.9	58							
	Middle Occipital Gyrus/Cuneus (L/R)	20	−84	8	3.9	731							
*Limbic Lobe*													
	Parahippocampal Gyrus (R)	38	−50	−8	3.3	58							
*Cerebellum*													
	Culmen/Fastigum (L/R)	−2	−54	−28	3.1	48							
	Declive (R)	12	−78	−26	3.1	51							
	Declive (R)	44	−72	−30	2.9	54							

### ROI Analysis

**Figure 2:**
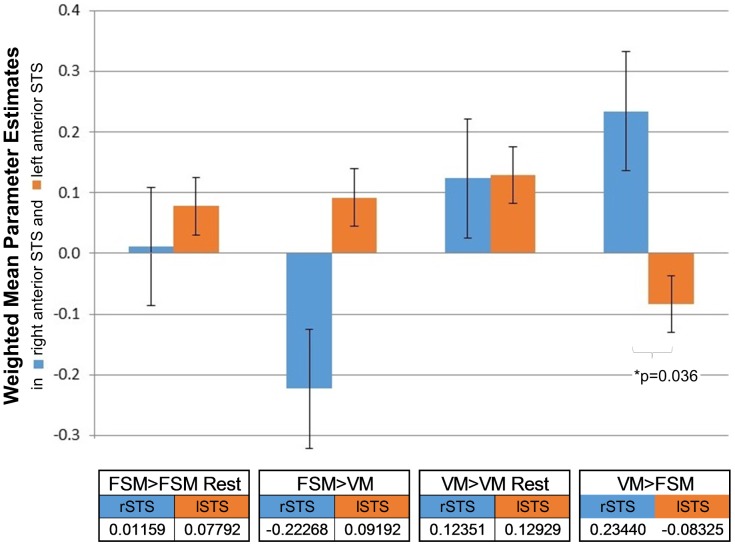
Weighted mean parameter estimates in right and left STS during voice or speech-sound directed information processing. Weighted mean parameter estimates extracted from regions of interest (*in blue*: right and *in orange*: left anterior STS) when focusing on speaker voice (‘VM>Rest’), speech sounds (‘FSM>Rest’), when focusing more on initial speech sounds of spoken object words than speaker voice (‘FSM>VM’) and when focusing more on speaker voice than the initial speech sounds of spoken object words (‘VM>FSM’; significant activation difference in right compared to left anterior STS with p = 0.036). Weighted mean parameter estimates as extracted from right (rSTS) and left anterior STS (lSTS) regions of interest are summarized below the bar graphic.

Since both VM and FSM elicited activation within bilateral superior temporal sulcus, we employed a region of interest analysis and further assessment of bilateral STS activations using a systematic approach as suggested by Bosch [60]. In a first step, we defined an independent right-hemispheric functional ROI (a region of interest was derived based on the right anterior STS in adults [15]) as well as a flipped left-hemispheric analogous ROI. In a second step, mean parameter estimates were extracted for these bilateral STS ROIs for the conditions ‘VM>Rest’, ‘FSM>Rest’, ‘FSM>VM’ and ‘VM>FSM’. There was significantly more activation during the speaker identification or voice matching task (‘VM>FSM’: mean parameter estimates  = 0.2) compared to the speech-sound specific, or first sound matching task (‘FSM>VM’: mean parameter estimates  = −0.2; p>0.01) within right STS. Finally, we employed a paired two-samples T-test to assess lateralization effects for voice identification (VM>FSM) in anterior STS regions [60] and observed a significance of p = 0.036, with the right anterior STS more strongly activated during voice identification compared to the left (see **Fig. 2** for a complete overview; Notably, mean parameter estimates for higher decimals reported are close to, but not exactly opposite, most likely because of subtle masking differences between the two contrasts). Since we here investigate a very young pediatric population, but have based our ROI analysis on adult coordinates ([15] due to a lack of studies in younger children), we further replicated our ROI findings using a right anterior STS region of interest based on activation from our second level contrast during voice matching (‘VM>FSM’) and achieved similar significant findings. We also performed a correlational analysis between behavioral measures and activity within our regions of interest to investigate the relationship between neuronal activation during voice matching and behavioral performance, however, we did not find any significant results.

### fcMRI Results

**Figure 3:**
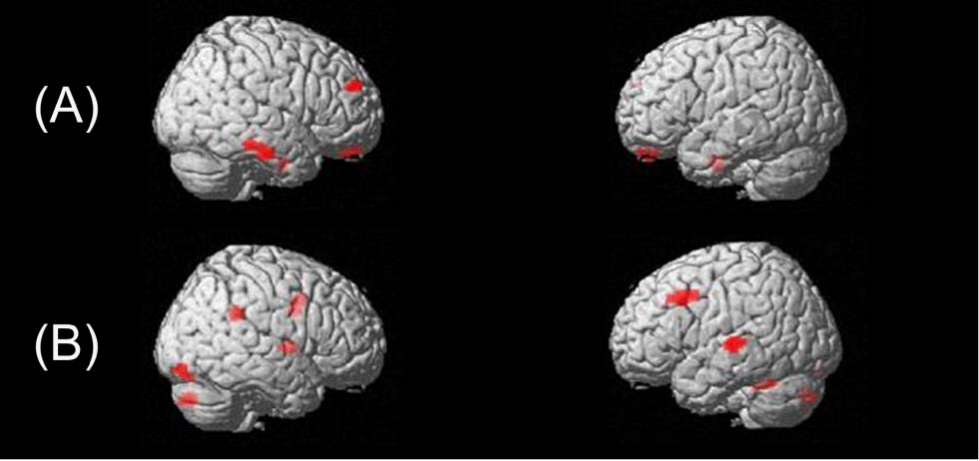
Functional connectivity maps during voice or speech-sound directed information processing. Statistical functional connectivity maps when attending to speakers voice (‘VM> FSM’) for (**A**) the left STS and (**B**) the right STS (p<0.005; k = 50).

We applied a post-hoc seed-to-voxel bivariate correlation analysis to explore networks of functionally connected regions with the seeds in the right and left STS as extracted for the ROI analysis. The seed-based analysis was performed for the contrast ‘VM>FSM’. Findings revealed positive correlations between right STS and left superior temporal gyrus (STG) and right-hemispheric supramarginal gyrus, middle frontal gyrus (MFG), putamen, middle occipital gyrus (MOG), cingulate gyrus and inferior frontal gyrus (IFG). For the left STS, we observed positive correlation with right-hemispheric superior frontal gyrus (SFG), postcentral gyrus and inferior temporal gyrus (ITG) (**Table 3**). **Fig. 3** shows the correlation maps for the left (**A**) and right (**B**) STS seeds (‘VM>FSM’).

**Table 3:** Main cortical regions that show connectivity with the two seed regions right STS (above) and left STS (below) in our connectivity analysis (‘VM>FSM’).

Seed Region	Target Region	x	y	z	Z	Size, voxels
						
**Right STS**						
	R subgyral	34	−42	30	4.55	87
	L middle frontal gyrus	−38	16	38	3.92	189
	L superior temporal gyrus	−64	−22	4	3.73	94
	R lentiform nucleus	24	−2	4	3.73	102
	R middle occipital gyrus	32	−84	−14	3.69	78
	R sub-gyral	20	6	38	3.63	101
	L cerebellum anterior lobe	−18	−52	−28	3.62	57
	R pyramis	14	−82	−38	3.27	99
**Left STS**						
	R superior frontal gyrus	42	48	26	4.05	75
	R rectal gyrus	6	50	−28	3.67	93

R = right; L = left.

## Discussion

The current paper investigates voice-specific compared to speech-sound specific processing in preschool-aged children. When compared to rest, both voice and speech-sound directed tasks activate bilateral primary and secondary auditory language areas (e.g., bilateral superior and middle temporal gyri), but also components of the language network (e.g., inferior frontal, temporoparietal and occipitotemporal brain regions). Focusing on the speaker's voice compared to speech-sound directed processing led to an increase in activation in the right middle/superior temporal gyri. Since there were no significant differences in in-scanner performance between voice matching and the first sound matching task, we conclude that the results cannot be accounted for by increased difficulty or varying attentional demands in either condition. Our findings therefore indicate that the human-specific voice region is already specialized by the age of five and is similar to that seen in typical adults (e.g., [9], [10], [15], [19], [22]).

Our results are in line with studies showing that the right STS is crucial for the extraction of the acoustic features related to voice recognition, similar to other speaker identification tasks [15], [61]–[64]. Neuroimaging research has repeatedly implicated brain regions along the STS during voice and speech processing incorporating linguistic and extra-linguistic information, such as speaker identity, in the human [3], [11] and non-human brain [65]. It has been suggested that understanding speech content involves a hierarchy of processes. For example, sound-based representations of speech particularly rely on bilateral posterior-superior temporal lobes, whereas meaning-based representations are more strongly represented within the left temporal-parietal-occipital junction [66]. Furthermore, voice recognition differs from the analysis of speech-sound specific content in that it requires a fine-tuned analysis of the vocal structure of speech [11]. Similar to the model of face perception, it has been proposed that the neuronal voice perception system may represent an ‘auditory face’ model [11]. Our findings are in favor of such a fine-grained auditory analysis of the human voice. However, it is important to note that the task used in the current study required participants to match voices based on speaker gender, a task that requires processing of the acoustic properties inherent to the voice of the speaker. These cues include the fundamental frequency of the speaker (pitch) as well as the vocal quality (i.e. timbre, or spectral formants), which often provide context cues as to whether the speaker is male or female. Similarly, basic pitch processing has been implicated in the right auditory cortex in the STS/STG and planum temporale [67]–[68]. Thus, since the present task does not require voice recognition per se, the activation of voice-specific brain regions may imply that the right anterior STS, along with right STS regions such as the planum temporale, processes acoustic voice identification features in general (such as pitch, vocal quality, and gender) in our age group.

Seed-based fcMRI findings suggest that the right and left STS are correlated with distinct functional networks during voice processing in pre-school children. While the right STS correlates positively with left-hemispheric STG and right-hemispheric temporal, occipital and frontal regions, the left STS correlates with different right-hemispheric frontal and temporal regions. Three previous studies investigating voice identity in adults have reported positive correlations between contralateral STS and STG [10], [44]–[45]. The observed positive correlations between the right STS and the right IFG and MFG in pre-school children are in line with findings reported in adults [44]–[45], which may suggest a higher cognitive involvement in voice identity matching based on individual vocal and glottal parameters. Thus, we suggest that functional correlations between the right STS and temporal/frontal regions during voice processing in pre-school children may be comparable to functional networks previously observed in adults. Finally, in line with our findings, both research groups reported positive correlations between the right STS and ipsilateral frontal regions such as the IFG [45] and the dorsolateral prefrontal cortex [44].

Notably, we employed a behavioral interleaved gradient design due to the nature of our auditory discrimination task. Others have previously demonstrated that functional networks can be observed by correlating sparse-sampled time-series data [93]–[94], [97]–[99]. Though not optimal for fcMRI analysis, this design is crucial for auditory experiments (e.g., in order to present auditory stimuli without interference from scanner background noises [89]–[93]), especially in the context of auditory selective attention. Scanner background noise (SBN) can increase BOLD signal in auditory and language regions resembling a task-induced hemodynamic response in a highly variable manner across subjects, and SBN during rest conditions can further mask or alter the BOLD signal in a non-linear fashion [57]. Since fcMRI is inherently more sensitive to non-neuronal sources of noise than traditional fMRI analysis, sparse temporal sampling may be warranted to avoid spurious correlations due to scanner background noise. Although we collected relatively fewer time-points with lower temporal resolution than typical of continuous scanning designs, Van Dijk and colleagues have shown that fcMRI is robust to long TRs [100]. Furthermore, the low-frequency fluctuations of interest in fcMRI (typically <0.1 Hz) should be captured within our 6 second TR, and we sampled a consistent number of time points across all conditions.

Bilateral superior temporal sulci have shown to be recruited for a wide range of pragmatic communicative tasks. Neuroimaging studies have implicated this brain region during tasks targeting theory of mind and mentalization [69]–[71], motion perception [72]–[73], person impressions [74], gestures [75], face [76] and speech perception [77] as well as social attention [76]. Because of the diversity of roles of the bilateral STS in social neuroimaging tasks, it has been argued that this region may be responsible for interpreting social communicative significance in general [78]. It has been hypothesized that the right STS may not be a voice-specific area in the human brain per se, but rather represents an area that is responsible for processing vocal sounds that are communicative in nature. For example, Shultz and colleagues [79] employed an fMRI task to demonstrate that neuronal activation within the right STS increases when presented with communicative vocal sounds (e.g., speech and laughter, see [80]) in comparison with non-communicative vocal sounds (e.g., coughing and sneezing) [79]. These findings are in line with our results (where first-sound matching represents a non-communicative and voice-matching a communicative task). Understanding the role of the STS in differentiating between communicative and non-communicative sounds may be critical regarding implications for disorders of social communication, such as ASD; disorders in which the region within the right STS has been found to be hypoactivated (e.g., [81]). In addition, individuals with social communication disorders show structural alterations in brain regions which again include bilateral brain areas of the STS (e.g., [82]–[83]).

Although we observed significant differences when comparing the processing of voice- specific and speech-sound directed speech stimuli within the right anterior STS, we acknowledge certain limitations. It is noteworthy that only one female and one male voice were used in this study. For example, it has been shown that female voices may produce stronger neuronal responses than male voices, despite a right hemispheric dominance in the STG for both male and female voices [7]. However, the use of male and female voices has been counterbalanced across our experimental conditions. Future studies should include a wide range of different speakers, particularly varying in gender, fundamental frequency, or age. Furthermore, the current study employed a voice matching task, which does not necessarily demand recognition of speaker voice. Thus, these findings reflect the neural mechanisms involved in processing communicative vocal sounds, but need to be interpreted carefully in relation to general processes required for voice recognition. An additional potential limitation of the current study is the absence of an adult participant control group. However, there is a robust body of existing research demonstrating which regions are recruited in adults when completing similar tasks [10] and activation peaks from these studies have been adapted and further studied here. Still, we cannot rule out that there are not differences in brain activation and functional connectivity between children and adults without an adult control group. Finally, due to the aforementioned temporal restrictions of our study design and the BOLD signal itself, our fcMRI results are not directly comparable to connectivity work employing other neuroimaging modalities such as EEG or MEG, and therefore should be interpreted with caution.

Impairments in speech perception or any of its related relevant features have been reported in various disorders of social communications or perception, including autism-spectrum disorder [14],[81], schizophrenia [84], Parkinson's disease and Alzheimer's disease [85]–[86], as well as in patients with acquired brain injuries, such as phonagnosia [12]–[13], ventral frontal lobe damage [87] and right hemispheric dysfunctions [88]. Understanding the behavioral and neural basis of these disorders first requires greater knowledge about speech processing in typically developing populations. Due to technical and practical challenges, few neuroimaging research studies include younger children and many conclusions about infants and children with developmental disorders are based on the assumption of a static adult brain [40]. However, modern neuroimaging tools, such as EEG, MRI and NIRS, offer the means for research targeting abnormal brain growth, development and function in pediatric populations (e.g [31]–[33]). We suggest that the current findings in typically developing children may be utilized to broaden understanding of neurophysiological findings in atypically developing children, particularly within disorders of social communication.

In conclusion, the present study demonstrates neuronal differences between the processing of voice versus speech-specific information in preschool-aged children within right anterior STS. Our findings indicate that the human-specific voice region within the right anterior STS is already specialized by the age of five and is similar to that seen in typical adults. Additionally, positive functional correlations between the right STS with left-hemispheric STG and right-hemispheric temporal, occipital and prefrontal regions were observed. Our findings may have implications within the fields of typical and atypical language and social development. In particular, this work may guide future studies investigating young children with speech impairments and disorders of social communication.

## Supporting Information

S1 Table **Socioeconomic Status (SES).**
(DOC)

S2 Table **Language Development.**
(DOC)
