# Supplementary material for: Investigating the Neural Correlates of Voice versus Speech-Sound Directed Information in Pre-School Children
Source: PLoS One. 2014 Dec 22;9(12):e115549. doi: 10.1371/journal.pone.0115549 (PMC4274095; doi:10.1371/journal.pone.0115549)
Supplement: S1 Table — Socioeconomic Status (SES). (DOC) [file pone.0115549.s001.doc]

| **S1.** Socioeconomic Status (SES) | | | | | |
| --- | --- | --- | --- | --- | --- |
|  | **N=18** (Two families did not fill out the questionnaire) | |  | |  |
|  |  |  |  | |  |
|  |  |  | [%] | |  |
|  |  |  |  |  |  |
|  | **Mother Characteristics (%)** |  |  | |  |
|  |  |  |  | |  |
|  | **Education (highest degree earned)** | High School Diploma/GED | 0.0% | |  |
|  |  | Associate Degree | 0.0% | |  |
|  |  | Bachelor's Degree | 55.5% | |  |
|  |  | Master's Degree | 33.3% | |  |
|  |  | Doctorate | 5.6% | |  |
|  |  | Professional(MD, JD) | 0.0% | |  |
|  |  | OtherSpecify | 0.0% | |  |
|  |  | No Response | 5.6% | |  |
|  |  |  |  |  |  |
|  | **Current activity** | Working Full Time | 22.2% | |  |
|  |  | Working Part Time | 16.7% | |  |
|  |  | Unemployed or laid off | 0.0% | |  |
|  |  | Looking for work | 0.0% | |  |
|  |  | Staying At Home, raising a child | 61.1% | |  |
|  |  | Retired | 0.0% | |  |
|  |  | No Response | 0.0% | |  |
|  |  |  |  | |  |
|  | **Money earned within the last 12 months** | Less Than $5000 | 44.4% | |  |
|  |  | $5,000-$11,999 | 5.6% | |  |
|  |  | $12,000-$15,999 | 0.0% | |  |
|  |  | $16,000-$24,999 | 0.0% | |  |
|  |  | $25,000-$34,999 | 0.0% | |  |
|  |  | $35,000-$49,000 | 5.6% | |  |
|  |  | $50,000-$74,999 | 16.7% | |  |
|  |  | $75,000-$99,999 | 5.6% | |  |
|  |  | $100,000 and Greater | 5.6% | |  |
|  |  | Don't know | 0.0% | |  |
|  |  | No Response | 16.7% | |  |
|  |  |  |  | |  |
|  | **Home owner status** | Home Rented for Money | 16.7% | |  |
|  |  | Home Owned By You | 83.3% | |  |
|  |  | Home Owner Status Not Available | 0.0% | |  |
|  |  |  |  | |  |
|  |  |  |  | |  |
|  | **Family Characteristics (%)** |  |  | |  |
|  |  |  |  |  |  |
|  | **Money earned within the last 12 months** | Less Than $5000 | 0.0% | |  |
|  |  | $5,000-$11,999 | 0.0% | |  |
|  |  | $12,000-$15,999 | 0.0% | |  |
|  |  | $16,000-$24,999 | 0.0% | |  |
|  |  | $25,000-$34,999 | 0.0% | |  |
|  |  | $35,000-$49,000 | 0.0% | |  |
|  |  | $50,000-$74,999 | 5.6% | |  |
|  |  | $75,000-$99,999 | 22.2% | |  |
|  |  | $100,000 and Greater | 61.1% | |  |
|  |  | Don't know | 0.0% | |  |
|  |  | No Response | 11.1% | |  |
|  | **Length of time you could maintain standard of living if all income is lost** |  |  |  |  |
|  |  | Less than 1 Month | 5.6% | |  |
|  |  | 1-2 Months | 11.1% | |  |
|  |  | 3-6 Months | 33.3% | |  |
|  |  | 7-12 Months | 33.3% | |  |
|  |  | More Than 1 Year | 11.1% | |  |
|  |  | No Response | 5.6% | |  |
|  |  |  |  |  |  |
