# Supplementary material for: Investigating the Neural Correlates of Voice versus Speech-Sound Directed Information in Pre-School Children
Source: PLoS One. 2014 Dec 22;9(12):e115549. doi: 10.1371/journal.pone.0115549 (PMC4274095; doi:10.1371/journal.pone.0115549)
Supplement: S2 Table — Language Development. (DOC) [file pone.0115549.s002.doc]

| **S2.** Language Development | | | | | | |
| --- | --- | --- | --- | --- | --- | --- |
|  | **N=19** (One family did not fill out the questionnaire) | | |  |  |  |
|  |  |  |  |  | |  |
|  |  |  |  | [%] | |  |
|  |  |  |  |  |  |  |
|  | **Language Development** |  |  |  | |  |
|  |  |  |  |  | |  |
|  | **To the best of your knowledge, when did your child produce his or her first word?** |  | 6-12 months | 52.6% | |  |
|  |  |  | 1-1.5 years | 36.8% | |  |
|  |  |  | 1.6-2 years | 5.3% | |  |
|  |  |  | 2-2.5 years | 0.0% | |  |
|  |  |  | 2.6-3 years | 0.0% | |  |
|  |  |  | Other | 0.0% | |  |
|  |  |  | No Response | 5.3% | |  |
|  |  |  |  |  | |  |
|  | **When did your child start producing sentences (3 words or more)?** |  | 1-1.5 years | 52.6% | |  |
|  |  |  | 1.6-2 years | 31.6% | |  |
|  |  |  | 2-2.5 years | 15.8% | |  |
|  |  |  | 2.6-3 years | 0.0% | |  |
|  |  |  | 3-3.5 years | 0.0% | |  |
|  |  |  | 3.6-4 years | 0.0% | |  |
|  |  |  | 4-4.5 years | 0.0% | |  |
|  |  |  | No Response | 0.0% | |  |
|  |  |  |  |  | |  |
|  | **When did your child start understanding simple verbal commands (like stop or give me)?** |  | 3-4 months | 0.0% | |  |
|  |  |  | 6-12 months | 73.7% | |  |
|  |  |  | 1-1.5 years | 26.3% | |  |
|  |  |  | 1.6-2 years | 0.0% | |  |
|  |  |  | No Response | 0.0% | |  |
|  |  |  |  |  | |  |
|  | **Has your child been officially diagnosed with a language disorder, reading disability, mood disorder or attention difficulties?** |  | Yes | 0.0% | |  |
|  |  |  | No | 100.0% | |  |
|  |  |  | No Response | 0.0% | |  |
|  |  |  |  |  |  |  |
|  |  |  |  |  | |  |
|  |  |  |  |  | |  |
